# Anticholinergic burden and clinical outcomes among older adults admitted in a tertiary hospital: a prospective cohort study

**DOI:** 10.1371/journal.pone.0332946

**Published:** 2025-09-19

**Authors:** Pornkamol Tiranaprakij, Sahaphume Srisuma, Krongtong Putthipokin, Sirasa Ruangritchankul

**Affiliations:** 1 Division of Geriatric Medicine, Department of Medicine, Faculty of Medicine Ramathibodi Hospital, Mahidol University, Bangkok, Thailand; 2 Division of Clinical Pharmacology and Toxicology, Department of Medicine, Faculty of Medicine, Ramathibodi Hospital, Mahidol University, Bangkok, Thailand; 3 Clinical Pharmacy Unit, Pharmacy Division, Faculty of Medicine Ramathibodi Hospital, Mahidol University, Bangkok, Thailand; Ekiti State University College of Medicine, NIGERIA

## Abstract

**Background:**

Anticholinergic medication use is associated with adverse clinical outcomes, especially in older adults. However, few studies have assessed the anticholinergic burden in the Thai geriatric population. Hence, we aimed to evaluate the impact of anticholinergic burden on clinical outcomes in older patients after discharge from the hospital.

**Methods:**

A prospective cohort study was conducted between January 1 to December 31, 2023. The prescribed medications were assessed at admission and discharge to determine the anticholinergic cognitive burden (ACB) scores. Participants were classified into three groups according to the ACB score at discharge: none (score 0), moderate (score 1–2), and severe (score ≥ 3) anticholinergic burden. The Cox proportional hazard model was used to determine the marker risk of high anticholinergic burden to adverse outcomes.

**Results:**

This study involved 290 older patients admitted to general internal medicine wards. At discharge, 37.9% (n = 110) of the patients had a high anticholinergic burden (ACB score ≥ 3), and 50% (n = 145) had a higher ACB score than at admission. The three most commonly prescribed anticholinergics at discharge were benzodiazepines (20.3%), corticosteroids (20.0%), and antihistamines (15.9%). During the one-year follow-up period, 16.6% (n = 48) of the patients died. The incidence rate of all-cause mortality in hospitalized older patients with an ACB score ≥ 3 was 0.65 cases per 1000-person day during a one-year follow-up period. After adjusting for potential factors, an ACB score of ≥ 3 at discharge was marginally associated with one-year mortality post discharge [hazard ratio: 2.98, 95% confidence interval (0.96–9.28)].

**Conclusions:**

The exposure to high anticholinergic burden (ACB scores ≥ 3) at discharge was slightly associated with an increased risk of one-year mortality post discharge. The cautious use of benzodiazepines may assist to reduce the anticholinergic burden in this vulnerable population.

## Introduction

The proportion of geriatric individuals has risen markedly worldwide over the past decade [1]. In Thailand, the number of people aged 60 and over was estimated to reach 12.6 million in 2023 and is predicted to be 16.7 million by 2040 [2]. According to the British Geriatric Society, older adults aged over 65 years are prone to be admitted to acute hospital care due to increased acute medical conditions and diseases, accounting for 60% of admitted patients [3].

The medical care provided to older patients consists of both non-pharmacological and pharmacological treatments. In clinical practice, several commonly prescribed pharmacological treatments in older adults have anticholinergic properties, such as antihistamines, tricyclic antidepressants, and antipsychotics [4–7]. These medications have been associated with numerous adverse clinical outcomes in the geriatric population, including increased risks of falls [8], reduced physical activity [9], cognitive impairment [10], and increased mortality [11]. The heightened susceptibility of older adults to the effects of anticholinergic medications is influenced by age-related changes in the nervous system, as well as alterations in pharmacokinetics and pharmacodynamics. According to age-related changes in the central nervous system (CNS), older adults experience decreased cholinergic neurons and receptors, which reduces baseline acetylcholine activity. Additionally, increased blood-brain barrier permeability permits more extensive penetration of anticholinergic drugs into the CNS, resulting in sedation, cognitive impairment, and delirium [5,12]. Regarding aging changes in the peripheral nervous system, older adults are more likely to have diminished autonomic nervous system reserve, which reduces their ability to compensate for parasympathetic blockade owing to anticholinergic drugs, leading to adverse effects such as urinary retention, dry mouth, and constipation [12,13]. These adverse effects on both the central and peripheral nervous systems contribute to higher rates of hospitalization and greater healthcare expenditure [14].

The prevalence of anticholinergic medication use among hospitalized geriatric patients varies significantly by country and anticholinergic burden measures. In England, it has been reported at 30.5% based on the Anticholinergic Risk Scale. In Poland, the prevalence is 40.7%, while in Germany, it reaches as high as 74.4%, according to the Anticholinergic Cognitive Burden (ACB) scale [15–17]. Various scales have been developed to quantify the anticholinergic burden in clinical practice, such as Anticholinergic Risk Scale, Anticholinergic Cognitive Burden scale, and Anticholinergic Drug Scale [18–20]. The ACB score is the most validated instrument in terms of its ability to determine the relationship between anticholinergic medication use and outcomes such as mortality [11,18,21,22]. Although there are several studies that evaluated inappropriate medications for Thai geriatric populations [23,24], no research has specifically investigated the relationship between anticholinergic burden and mortality in older Thai adults. The authors anticipate that the findings of this study will enhance physicians’ awareness of anticholinergic medication use in clinical practice.

Therefore, the primary objective of the study was to evaluate the impact of severe anticholinergic burden (ACB score ≥3) on clinical outcomes such as mortality, rehospitalization, emergency department (ED) revisits, and falls in the year after discharge from a tertiary hospital. The second objective was to analyze the incidence rates of adverse clinical outcomes one year after discharge in patients with a high ACB score and assess changes in the ACB score from admission to discharge in geriatric patients in a Thai tertiary care center.

## Materials and methods

### Study design, setting and participants

The prospective cohort study was conducted in internal medicine wards at Ramathibodi Hospital, Mahidol University. The older patients aged 60 years and older consecutively admitted to internal medicine wards between January 1 and December 31, 2023, were enrolled in the study. All eligible participants had a hospital stay longer than 48 hours and were followed up on clinical outcomes for one year after discharge. Adverse clinical outcomes were recorded at 1, 3, 6, and 12 months post discharge by reviewing the patients’ electronic medical records (EMRs). Patients who were unable to communicate and provide informed consent or had a terminal illness with a life expectancy of < 3 months were excluded from the study. Additionally, patients who were subsequently transferred to the intensive care unit or lost to follow-up after discharge, or died in the hospital were excluded as well.

The sample size was calculated using the n4studies sample size application [25]. According to the previous data [11], presenting a high ACB score was associated with mortality of a type I error of 0.05, a type II error of 0.2, the exposed proportion of 0.31, the unexposed proportion of 0.17, the sample size to be collected was 290. The total ACB score was calculated using the summation of the assigned ACB value on a scale from 0 to 3 based on anticholinergic burden [19]. In the study, the participants were classified into three groups based on their anticholinergic burden at discharge: no anticholinergic burden (ACB score: 0), moderate anticholinergic burden (ACB score: 1–2), and severe anticholinergic burden (ACB score: ≥ 3).

**Fig 1:**
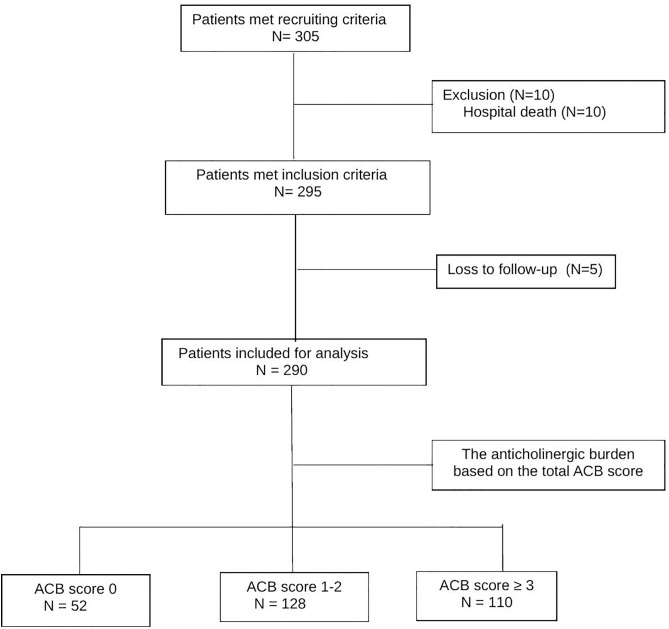
Flow chart of sample recruitment.

During the inclusion period, 305 potential participants were admitted to internal medicine wards. Among these patients, 10 were excluded due to hospital mortality, leaving 295 participants who fulfilled the inclusion criteria. Additionally, five patients were lost to follow-up during the study period. Therefore, the data analysis included the remaining 290 older hospitalized individuals, as illustrated in Fig 1.

### Data collection and measurement tools

The individual data were obtained from 290 older patients admitted to internal medicine wards at Ramathibodi Hospital, Mahidol University, from January 1 to December 31, 2023. The comprehensive data were retrieved from interview-administered questionnaires, electronic medical records, and clinical assessments. Within 48 hours of admission, trained medical staff gathered information on each patient’s baseline and clinical characteristics, biochemical parameters, and pharmacological therapy. Medical personnel assessed each patient’s clinical and functional status and conducted face-to-face interviews through questionnaires.

#### EMR information.

The data obtained from the EMRs included information on the patient’s demographic attributes such as sex and age; health status: primary diagnosis, chronic illnesses, Charlson comorbidity Index (CCI), and psychiatric disorders; body weight and height; currently prescribed medications; healthcare service utilization: length of hospital stays (LOS), visits to the ED and outpatient department (OPD), and hospital admissions within preceding 12 months. Information on biochemical parameters relevant to anticholinergic burden was obtained from EMRs as well. The records also documented all lists of prescribed medications at admission and discharge. All medications were documented and reported according to the codes established by the Anatomical Therapeutic Chemical (ATC) classification system, which served as a standard system endorsed by the World Health Organization [26]. Polypharmacy and excessive polypharmacy, defined as the concurrent use of five or more medications and ten or more medications, respectively, were assessed as potential risk factors for high anticholinergic burden [27–29].

#### Face-to-face interview-administered questionnaire.

Within the first 48 hours after admission, face-to-face interviews were conducted alongside administered questionnaires to collect data on socio-demographic profiles, geriatric syndrome, psycho-cognitive status, and functional capacity. The structured interviews were performed to gather information on sociodemographic profiles, such as educational attainment, living and marital status, medical insurance coverage, and caregiver arrangements. In addition, medical staff collected data on patients’ lifestyle factors, including smoking status, alcohol consumption, and herbal product and substance use.

They also assessed the presence of various geriatric syndromes, namely visual and auditory impairment, pressure ulcers, incontinence, insomnia, falls, and impaired activity of daily living (ADL) [30–32]. The individual’s functional capacity was evaluated using the modified Barthel ADL index in Thai version [33] and the Lawton instrumental activity of daily living (IADL) index [34]. The Barthel ADL index, rated from 0 to 20, assesses essential abilities on incontinence, feeding, bathing, ambulation, dressing, self-grooming, and toileting. Lower scores indicate a higher degree of dependence [33]. The Lawton IADL index measures proficiency across eight complex tasks: shopping, financial and medication management, telephone use, transportation, food preparation, household chores, and laundry [34]. The cumulative scores established a scale ranging from 0 to 8, with higher scores indicating an enhanced level of functional capacity.

All participants underwent a cognitive function assessment using the Montreal Cognitive Assessment (MoCA) test, which is used to evaluate higher cortical function [35,36]. The summed MoCA score ranges from 0 to 30, with lower scores indicating worse cognitive function and a score < 25 reflecting cognitive impairment. Additionally, depression was assessed by qualified personnel using the Thai Geriatric Depression Scale-15 (TGDS-15), which ranges from 0 to 15. A TGDS-15 score of 6 or higher is indicative of depression [37,38].

#### Clinical assessments.

Within 48 hours of admission, all participants were informed to measure their body weight and height, and body mass index (BMI) [39]. BMI is defined as an individual’s body weight in kilograms divided by the square of their height in meters [39]. Additionally, experienced medical staff evaluated each patient’s risk of malnutrition using the Nutritional Alert Form (NAF) [40], which consisted of eight sections designed to BMI, body composition, dietary intake and patterns, weight change, chewing and swallowing difficulties, medical conditions associated with malnutrition, functional capacity, serum albumin concentration, and lymphocyte count. The high score of NAF indicated a more severe level of malnutrition, categorized into scores of 0–5 (normal-mild malnutrition), 6–10 (moderate malnutrition), and ≥ 11 (severe malnutrition) [40]. The Clinical Frailty Scale (CFS) [41] was used to assess frailty in this aging population. The CFS has a range of 1 (robust) to 9 (terminally ill) [42], and patients with a score ≥ 5 are considered frail [41].

#### Medication exposures.

Each individual’s anticholinergic burden was calculated using the ACB scale [19]. The list of medications prescribed at the time of admission was retrieved from a medication reconciliation flowsheet by clinical pharmacists and physicians. Additionally, external medication lists and records of previous medication use were obtained from patients and caregivers and shared with nurses and medical staff. The list of medications prescribed at discharge was extracted from the EMR discharge summary. We gathered data on regular drugs only. In this study, we used the ACB score to assess anticholinergic burden. The ACB score identified the exposure to medication with anticholinergic activities. Medications with no anticholinergic effects were scored as 0. Medications with possible anticholinergic activities but with no clinically relevant negative cognitive effects were scored as 1. Medications with definite anticholinergic properties and clinically relevant cognitive effects were scored as 2 or 3, as shown in S1 Table. The total ACB was calculated using the summation of the assigned ACB value on a scale from 0 to 3 based on anticholinergic burden [19]. In the study, patients were categorized into one of three categories based on the total ACB score at the time of discharge: no ACB (score 0), moderate ACB (score 1–2), and severe ACB (score ≥ 3). This classification is considered clinically relevant such as cognitive impairment and mortality, according to previous studies [20,43,44].

#### Outcomes.

The primary outcome was the relationship between the ACB score and all-cause mortality one year after discharge. The secondary outcomes were the association of high ACB score with unexpected rehospitalization, ED revisits, and falls within a year after discharge. These clinical adverse outcomes were retrieved at 1, 3, 6, and 12 months post discharge from the patients’ EMRs.

### Statistical analysis

All data were analyzed using the SPSS for Windows Software Package, Version 25 (SPSS Inc., Chicago, Ill., USA). The categorical data were presented as the percentage to describe demographic and clinical characteristics, whereas the continuous data were presented as mean ± standard deviation (SD) or median ± interquartile range (IQR). To compare the clinical and biochemical parameters between three groups, Pearson’s chi-square test was used for categorical variables, one-way analysis of variances (ANOVA) with the Tukey post hoc test was performed for normally distributed continuous data, and Kruskal–Wallis test was used for non-normally distributed data. Then, the multivariable Cox proportional hazards model was performed to estimate crude and adjusted hazard ratios (HRs) and 95% confidence intervals (CIs) for clinical outcomes one year after discharge, such as all-cause mortality, rehospitalizations, ED revisits, and falls for each group. The level of statistical significance was set at p < 0.05. The multivariable models were adjusted by the MoCA, NAF, CFS and CCI scores, age, sex, and number of prescribed medications to explore the risk of high anticholinergic burden to adverse clinical outcomes.

### Ethical considerations

The current study was approved by the Committee on Human Rights Related to Research Involving Human Subjects, Faculty of Medicine Ramathibodi Hospital, Mahidol University (protocol number: COA. MURA 2022/696) on November 30, 2022. This prospective cohort study was reported according to the guideline of the Strengthening the Reporting of Observational Studies in Epidemiology (STROBE) statement [45]. The study was conducted in accordance with relevant guidelines and regulations in the Declaration of Helsinki. All subjects were informed regarding the study’s purpose, process, and procedure. Then, written informed consent was obtained from each participant before the beginning date of the study.

## Results

### Baseline clinical characteristics and biochemical parameters

The participants were classified into three groups according to their total ACB score at discharge: ACB score 0 (n = 52, 18.0%), ACB score 1–2 (n = 128, 44.1%) and ACB score ≥ 3 (n = 110, 37.9%). The average age of the participants was 73.9 (SD 8.8) years, ranging from 60 to 100 years. The majority of the patients were female (53.4%) and had an education level of ≤12 years (61%). The minority were single (6%) or lived alone (1%).

The demographic characteristics of the participants were compared and analyzed based on the total ACB score at discharge, as detailed in Table 1. No significant differences were observed in age, sex, marital status, and educational level among the three groups (p > 0.05). Participants exposed to ACB score ≥ 3 were more likely to be discharged to residential aged care facilities than those with an ACB score of 0 (p = 0.017). With regard to lifestyle behaviors, the prevalence of herbal product use was substantially higher among the participants with an ACB score of 1–2 compared to the other groups (p = 0.035). In contrast, there were no significant differences in the prevalence of smoking or alcoholic consumption among the three groups (p > 0.05). In terms of healthcare services, individuals with an ACB score ≥ 3 had a remarkably higher frequency of ≥ 10 OPD visits within one year compared to those with lower ACB scores (p < 0.001). The median LOS did not differ significantly among the three groups (p = 0.150).

**Table 1:** Baseline characteristics among hospitalized older patients stratified by the total ACB score.

Characteristics	Total (n = 290) N (%)	ACB score at discharge			
		ACB score 0 N = 52 (18.0%) N (%)	ACB score 1–2 N = 128 (44.1%) N (%)	ACB score ≥ 3 N = 110 (37.9%) N (%)	*P* value
Age in years, mean (SD)	73.9 (8.8)	75.4 (7.3)	73.7 (9.1)	73.5 (9.1)	0.239^#^
Female	155 (53.4)	31 (59.6)	69 (53.9)	55 (50)	0.514^*^
Male	135 (46.6)	21 (40.4)	59 (46.1)	55 (50.0)	
**Educational status**					
≤12 years	177 (61.0)	28 (53.8)	78 (60.9)	71 (64.5)	0.427^*^
>12 years	113 (39.0)	24 (46.2)	50 (38.1)	39 (35.5)	
**Marital status**					
Single	17 (5.9)	2 (3.8)	9 (7.0)	6 (5.5)	0.693^*^
Married, widowed, or separated	273 (94.1)	50 (96.2)	119 (93.0)	104 (94.5)	
**Lifestyle**					
Drinking	33 (11.4)	5 (9.6)	19 (14.8)	9 (8.2)	0.247^*^
Smoking	58 (20.0)	11 (21.2)	28 (21.9)	19 (17.3)	0.658^*^
Herbal use	33 (11.4)	3 (5.8)	21 (16.4)	9 (8.2)	0.035^*^
**Socioeconomic status**					
Living alone	3 (1.0)	1 (1.9)	1 (0.8)	1 (0.9)	0.587^*^
Discharge to residential age care facility	61 (21.0)	4 (7.7)	27 (21.1)	30 (27.3)	0.017^*^
No caregiver	58 (20.0)	14 (26.9)	25 (19.5)	19 (17.3)	0.352^*^
Self-drug administration	128 (44.1)	25 (48.1)	56 (43.8)	47 (42.7)	0.809^*^
**Healthcare services**					
Self-paid	68 (23.4)	11 (21.2)	29 (22.7)	28 (25.5)	0.801^*^
History of OPD visits ≥10 within 1 year	196 (67.6)	23 (44.2)	93 (72.7)	80 (72.7)	<0.001^*^
History of hospital admission ≥ 2 within 1 year	50 (17.2)	6 (11.5)	24 (18.8)	20 (18.2)	0.482^*^
History of ED visit ≥ 2 within 1 year	89 (30.7)	10 (19.2)	39 (30.5)	40 (36.4)	0.087^*^
LOS, median (IQR)	7.5 (5, 14)	6.5 (3.5, 11.5)	7 (5, 12.5)	10 (5, 15)	0.150^+^

**Data are presented as** mean (standard deviation), n (%), or median (interquartile range).

*Chi-square test, ^#^ Student’s t-test, + Mann–Whitney U test.

**Abbreviations:** ACB, anticholinergic cognitive burden; SD, standard deviation; IQR, interquartile range; ED, emergency department; OPD, outpatient department; LOS, length of stay.

With regard to clinical characteristics, septicemia (12.4%), myocardial infarction (7.6%), and urinary tract infection (6.2%) were the three leading causes of hospital admission. The most common chronic diseases were hypertension (73.8%), followed by dyslipidemia (60.7%), and diabetes mellitus (38.6%). As shown in Table 2, the number of comorbidities and the CCI were not significantly correlated with higher ACB scores. We also found that the patients exposed to an ACB score ≥ 3 were more likely to have dementia, anemia, and congestive heart failure than those with lower ACB scores (p = 0.04, p = 0.001 and p = 0.037, respectively). Nevertheless, the prevalence of cirrhosis or chronic kidney disease did not significantly differ among the groups.

**Table 2:** Clinical characteristics among hospitalized older patients stratified by the total ACB score.

Characteristics	Total (n = 290) N (%)	ACB score at discharge			
		ACB score 0 N = 52 (18.0%) N (%)	ACB score 1–2 N = 128 (44.1%) N (%)	ACB score ≥ 3 N = 110 (37.9%) N (%)	*P* value
**Comorbidities**					
No. of chronic diseases, median (IQR)	5 (4, 6)	4 (3, 6)	5 (3, 6)	5 (4, 6)	0.121^+^
CCI, median (IQR)	6 (4, 8)	5 (4, 7)	6 (4, 8)	6 (4, 8)	0.456^+^
CCI score ≥ 3	258 (89.0)	45 (86.5)	111 (86.7)	102 (92.7)	0.279^*^
Dyslipidemia	176 (60.7)	33 (63.5)	76 (59.4)	67 (60.9)	0.877^*^
Coronary artery disease	75 (25.9)	13 (25.0)	29 (22.7)	33 (30.0)	0.430^*^
Congestive heart failure	48 (16.6)	3 (5.8)	21 (16.4)	24 (21.8)	0.037^*^
Arrhythmia	48 (16.6)	6 (11.5)	21 (16.4)	21 (19.1)	0.482^*^
Hypertension	214 (73.8)	38 (73.1)	94 (73.4)	82 (74.5)	0.973^*^
Cirrhosis	18 (6.2)	4 (7.7)	7 (5.5)	7 (6.4)	0.851^*^
COPD	27 (9.3)	6 (11.5)	14 (10.9)	7 (6.4)	0.399^*^
Osteoarthritis	12 (4.1)	3 (5.8)	3 (2.3)	6 (5.5)	0.391^*^
Diabetes mellitus	112 (38.6)	17 (32.7)	56 (43.8)	39 (35.5)	0.256^*^
Chronic kidney disease	90 (31.0)	14 (26.9)	34 (26.6)	42 (38.2)	0.121^*^
Anemia	97 (33.4)	17 (32.7)	29 (22.7)	51 (46.4)	0.001^*^
Gout	27 (9.3)	4 (7.7)	14 (10.9)	9 (8.2)	0.695^*^
Dementia	11 (3.8)	0 (0)	3 (2.3)	8 (7.3)	0.040^*^
Cerebrovascular disease	48 (16.6)	10 (19.2)	21 (16.4)	17 (15.5)	0.832^*^
Hypothyroidism	7 (2.4)	2 (3.8)	3 (2.3)	2 (1.8)	0.733^*^
Malignancy	62 (21.4)	10 (19.2)	31 (24.2)	21 (19.1)	0.577^*^
Thromboembolism	11 (3.8)	1 (1.9)	4 (3.1)	6 (5.5)	0.475^*^
Vitamin D deficiency	27 (9.3)	6 (11.5)	8 (6.3)	13 (11.8)	0.280^*^
Systemic Lupus Erythematosus	4 (1.4)	0 (0)	2 (1.6)	2 (1.8)	1.000^*^
Rheumatoid arthritis	3 (1.0)	0 (0)	2 (1.6)	1 (0.9)	1.000^*^

**Data are presented as** n (%), or median (interquartile range).

*Chi-square test, + Mann–Whitney U test.

**Abbreviations:** IQR, interquartile range; COPD, chronic obstructive pulmonary disease; CCI, Charlson Comorbidity Index; ACB, anticholinergic cognitive burden.

Additionally, there was no significant difference in the proportion of geriatric syndromes, such as urinary and fecal incontinence, falls, pressure ulcers, hearing and visual impairments, sleep problems, weight loss, and dependency status, across the three groups (p > 0.05), as shown in S2 Table. Regarding functional abilities, the median IADL score demonstrated a remarkable decline with an increase in the total ACB score (p = 0.021). In contrast, the median MoCA, TGDS-15, and NAF scores and the mean Barthel ADL and CFS scores were not substantially different among the groups (p > 0.05).

Table 3 illustrates the changes in anticholinergic drug usage and the anticholinergic burden from admission to discharge. The results showed that the number of medications used at discharge was significantly higher than the number used at admission [11 items (IQR 8–14 items) vs 8 items (IQR 5–11 items), respectively]. Moreover, there was a higher proportion of patients with polypharmacy and excessive polypharmacy at discharge than at admission (polypharmacy: 96.6% vs 78.6% and excessive polypharmacy: 67.6% vs 34.8%). The proportion of patients prescribed at least one anticholinergic medication was 65.2% (n = 189) at admission and 82.1% (n = 238) at discharge. The median ACB score at admission was 1 (IQR 0–2). The ACB score at discharge ranged from 0 to 10, with the median ACB score at discharge was 2 (IQR 1–3). The results indicated that 21% (n = 61) of the patients had an ACB score ≥ 3 at admission compared to 37.9% (n = 110) at discharge. In addition, 50% (n = 145) of the patients showed increased use of anticholinergic medications, and 51.4% (n = 149) had higher ACB scores at discharge compared to admission. At discharge, the most frequently used anticholinergic medications were benzodiazepines (20.3%). The list of all anticholinergic medicines at admission and discharge are shown in S3 and S4 Tables, respectively.

**Table 3:** The changes in medication profiles among hospitalized older patients from admission to discharge.

Characteristics	Admission (n = 290) N (%)	Discharge (n = 290) N (%)
Number of prescribed medication per person, median (IQR)	8 (5, 11)	11 (8, 14)^+^
Polypharmacy	228 (78.6)	280 (96.6)^*^
Excessive polypharmacy	101 (34.8)	196 (67.6)^*^
Patients exposed to anticholinergics	189 (65.2)	238 (82.1)^*^
Number of anticholinergics per person, median (IQR)	1 (0, 2)	2 (1, 3)^+^
The total ACB score per person, median (IQR)	1 (0, 2)	2 (1, 3)^+^
Patients exposed to ACB score ≥ 3	61 (21)	110 (37.9)^*^
**Common anticholinergic drugs**		
Benzodiazepines	37 (12.8)	59 (20.3)^*^
Corticosteroids	43 (14.8)	58 (20.0)^*^
Antihistamines	31 (10.7)	46 (15.9)^*^

**Data are presented as** n (%) or median (interquartile range)

*Chi-square test, + Mann–Whitney U test

**Abbreviations:** ACB, anticholinergic cognitive burden; IQR, interquartile range

All prescribed medications at discharge were categorized using the ATC classification system, as shown in Table 4. Patients with an ACB score ≥ 3 were prescribed a significantly higher median number of medications than patients with lower ACB scores (p < 0.001). Furthermore, among the patients with an ACB score ≥ 3, the proportion of patients exposed to polypharmacy and excessive polypharmacy was remarkably higher than among those with an ACB score of 0 (p = 0.002 and p < 0.001, respectively). The prescription of antidiabetics, anticoagulants, vasoprotectives, antihypertensives, diuretics, beta-blocking agents, immunosuppressants, muscle relaxants, analgesics, psycholeptics, psychoanaleptics, cough and cold preparations, and antihistamines was significantly greater in the patients with an ACB score ≥ 3 compared to those with lower ACB scores (p < 0.05). The biochemical parameters of the patients were analyzed and revealed in S5 Table. There were no substantial differences in the biochemical profiles, including liver and kidney function among the three groups (p > 0.05).

**Table 4:** Prescribed medications use among hospitalized older patients stratified by the total ACB score.

Characteristics	Total (n = 290) N (%)	ACB score at discharge			
		ACB score 0 N = 52 (18.0%) N (%)	ACB score 1–2 N = 128 (44.1%) N (%)	ACB score ≥ 3 N = 110 (37.9%) N (%)	*P* value
Number of prescribed medications per person, median (IQR)	11 (8, 14)	8 (6, 10)	11 (9, 13)	14 (11, 16)	<0.001^+^
Polypharmacy	280 (96.6)	46 (88.5)	126 (98.4)	108 (98.2)	0.002^*^
Excessive polypharmacy	196 (67.6)	20 (38.5)	82 (64.1)	94 (85.5)	<0.001^*^
**Prescribed medications according to ATC classes and codes**					
A02 Drugs for acid related	185 (63.8)	28 (53.8)	79 (61.7)	78 (70.9)	0.087^*^
A06 Drugs for constipation	160 (55.2)	32 (61.5)	64 (50.0)	64 (58.2)	0.267^*^
A10 Drug used in diabetes	91 (31.4)	10 (19.2)	49 (38.3)	32 (29.1)	0.036^*^
A 11 Vitamins	185 (63.8)	27 (51.9)	87 (68.0)	71 (64.5)	0.125^*^
A 12 Mineral supplements	146 (50.3)	23 (44.2)	63 (49.2)	60 (54.5)	0.445^*^
B01 Antithrombotic agents	135 (46.6)	22 (42.3)	54 (42.2)	59 (53.6)	0.167^*^
B01A Anticoagulants	34 (11.7)	2 (3.8)	10 (7.8)	22 (20.0)	0.002^*^
C01 Cardiac therapy	15 (5.2)	5 (9.6)	0 (0)	10 (9.1)	0.002^*^
C05 Vasoprotectives	29 (10.0)	0 (0)	9 (7.0)	20 (18.2)	<0.001^*^
C02 Antihypertensives	64 (22.1)	2 (3.8)	25 (19.5)	37 (33.6)	<0.001^*^
C02CA Alpha-blockers	35 (12.1)	2 (3.8)	12 (9.4)	21 (19.1)	0.010^*^
C02DB02 Hydralazine	48 (16.6)	0 (0)	20 (15.6)	28 (25.5)	<0.001^*^
C03 Diuretics	50 (17.2)	1 (1.9)	21 (16.4)	28 (25.5)	0.001^*^
C07 Beta blocking agents	119 (41.0)	14 (26.9)	50 (39.1)	55 (50.0)	0.017^*^
C08 Calcium channel blockers	138 (47.6)	21 (40.4)	64 (50.0)	53 (48.2)	0.498^*^
C09 Agents acting on the renin angiotensin system	73 (25.2)	10 (19.2)	36 (28.1)	27 (24.5)	0.452^*^
C10 Lipid modifying agents	172 (59.3)	31 (59.6)	74 (57.8)	67 (60.9)	0.742^*^
H02 Corticosteroids	57 (19.7)	0 (0)	21 (16.4)	36 (32.7)	<0.001^*^
J01 Antibacterial drugs	129 (44.5)	25 (48.1)	60 (46.9)	44 (40.0)	0.481^*^
J05 Antivirals for systemic use	48 (16.6)	6 (11.5)	24 (18.8)	18 (16.4)	0.497^*^
L01 Antineoplastic agents	3 (1.0)	1 (1.9)	2 (1.6)	0 (0)	0.387^*^
L04 Immunosuppressants	25 (8.6)	0 (0)	9 (7.0)	16 (14.5)	0.006^*^
M03 Muscle relaxant	15 (5.2)	2 (3.8)	2 (1.6)	11 (10.0)	0.012^*^
N02 Analgesics	154 (53.1)	22 (42.3)	63 (49.2)	69 (62.7)	0.026^*^
N02A Opioids	36 (12.4)	0 (0)	11 (8.6)	25 (22.7)	<0.001^*^
N02BF Gabapenoids	42 (14.5)	5 (9.6)	12 (9.4)	25 (22.7)	0.008^*^
N03 Antiepileptics	19 (6.6)	1 (1.9)	8 (6.3)	10 (9.1)	0.223^*^
N05 Psycholeptics	91 (31.4)	3 (5.8)	31 (24.2)	57 (51.8)	<0.001^*^
N05A Antipsychotics	33 (11.4)	0 (0)	1 (0.8)	32 (19.1)	<0.001^*^
N05B-N05C Anxiolytics, sedatives and hypnoyics	60 (20.7)	1 (1.9)	28 (21.9)	31 (28.2)	0.001^*^
N06 Psychoanaleptics	32 (11.0)	0 (0)	13 (10.2)	19 (17.3)	0.004^*^
N06A Antidepressants	27 (9.3)	0 (0)	10 (7.8)	17 (15.5)	0.005^*^
N06D Anti-dementia drugs	10 (3.4)	0 (0)	4 (3.1)	6 (5.5)	0.199^*^
R03 Drugs for obstructive airway diseases	52 (17.9)	5 (9.6)	28 (21.9)	19 (17.3)	0.147^*^
R05 Cough and cold preparations	67 (23.1)	5 (9.6)	26 (20.3)	36 (32.7)	0.003^*^
R06 Antihistamines for systemic use	43 (14.8)	3 (5.8)	16 (12.5)	24 (21.8)	0.017^*^

**Data are presented as** n (%) or median (interquartile range).

* Chi-square test, + Mann–Whitney U test.

**Abbreviations:** IQR, interquartile range; ACB, anticholinergic cognitive burden.

### Clinical outcomes of anticholinergic burden

Tables 5 and 6 illustrate adverse clinical outcomes associated with anticholinergic burden, which included all-cause mortality, unplanned rehospitalization, unexpected ED visits, and falls. The prevalence of all-cause mortality at one year after discharge showed a minimal increase as the ACB score increased (ACB score of 0: 7.7%, ACB score of 1–2: 17.2%, ACB score ≥ 3: 20.0%; *p *= 0.139). Additionally, the incidence rates of all-cause mortality, falls, unexpected rehospitalization, and unplanned ED revisits one year after discharge marginally increased as the ACB score increased. The incidence rate for all-cause mortality at one year after discharge among the patients with an ACB score ≥ 3 was 0.65 cases per 1000 person-days.

**Table 5:** Adverse clinical outcomes after discharge among hospitalized older patients stratified by the total ACB score.

Characteristics	Total (n = 290) N (%)	ACB score at discharge			
		ACB score 0 N = 52 (18.0%) N (%)	ACB score 1–2 N = 128 (44.1%) N (%)	ACB score ≥ 3 N = 110 (37.9%) N (%)	*P* value
**1 month after discharge**					
All-cause mortality	8 (2.8)	0 (0)	6 (4.7)	2 (1.8)	0.214
Unexpected rehospitalization	42 (14.5)	5 (9.6)	23 (18.0)	14 (12.7)	0.283
Unexpected ED revisit	76 (26.2)	7 (13.5)	38 (29.7)	31 (28.2)	0.067
Fall	2 (0.7)	0 (0)	2 (1.6)	0 (0)	0.664
**3 months after discharge**					
All-cause mortality	17 (5.9)	1 (1.9)	10 (7.8)	6 (5.5)	0.305
Unexpected rehospitalization	81 (27.9)	10 (19.2)	40 (31.3)	31 (28.2)	0.265
Unexpected ED revisit	116 (40)	13 (25)	55 (43)	48 (43.6)	0.051
Fall	5 (1.7)	1 (1.9)	3 (2.3)	1 (0.9)	0.723
**6 months after discharge**					
All-cause mortality	38 (13.1)	3 (5.8)	19 (14.8)	16 (14.5)	0.223
Unexpected rehospitalization	107 (36.9)	17 (32.7)	47 (36.7)	43 (39.1)	0.732
Unexpected ED revisit	136 (46.9)	19 (36.5)	58 (45.3)	59 (53.6)	0.112
Fall	8 (2.8)	1 (1.9)	4 (3.1)	3 (2.7)	1.000
**12 months after discharge**					
All-cause mortality	48 (16.6)	4 (7.7)	22 (17.2)	22 (20.0)	0.139
Unexpected rehospitalization	139 (47.9)	23 (44.2)	60 (46.9)	56 (50.9)	0.693
Unexpected ED revisit	166 (57.2)	26 (50.0)	72 (56.3)	68 (61.8)	0.349
Fall	12 (4.1)	3 (5.8)	5 (3.9)	4 (3.6)	0.797

**Data are presented as** n (%).

Chi-square test.

**Abbreviations:** ED, emergency department; ACB, anticholinergic cognitive burden.

**Table 6:** Incidence rate of adverse clinical outcomes among hospitalized older patients stratified by the ACB score.

Characteristics	Total (n = 290) Cases/1000 person-days	ACB score at discharge		
		ACB score 0 N = 52 (18.0%) Cases/1000 person-days	ACB score 1–2 N = 128 (44.1%) Cases/1000 person-days	ACB score ≥ 3 N = 110 (37.9%) Cases/1000 person-days
**1 month after discharge**				
All-cause mortality	0.93	0	1.59	0.61
Unexpected rehospitalization	5.19	3.36	6.02	4.50
Unexpected ED revisit	10.34	4.88	12.06	11.23
Fall	0.23	0	0.53	0
**3 months after discharge**				
All-cause mortality	0.67	0.21	0.91	0.62
Unexpected rehospitalization	3.81	2.43	4.39	3.83
Unexpected ED revisit	6.23	3.26	7.01	7.01
Fall	0.19	0.22	0.28	0.10
**6 months after discharge**				
All-cause mortality	0.81	0.33	0.94	0.89
Unexpected rehospitalization	2.87	2.31	2.99	3.04
Unexpected ED revisit	4.22	2.73	4.31	5.02
Fall	0.17	0.11	0.20	0.17
**12 months after discharge**				
All-cause mortality	0.53	0.22	0.58	0.65
Unexpected rehospitalization	2.19	1.78	2.23	2.37
Unexpected ED revisit	3.06	2.14	3.14	3.53
Fall	0.14	0.17	0.14	0.12

**Data are presented as** cases/1000 person-days.

**Abbreviations:** ED, emergency department; ACB, anticholinergic cognitive burden.

**Fig 2:**
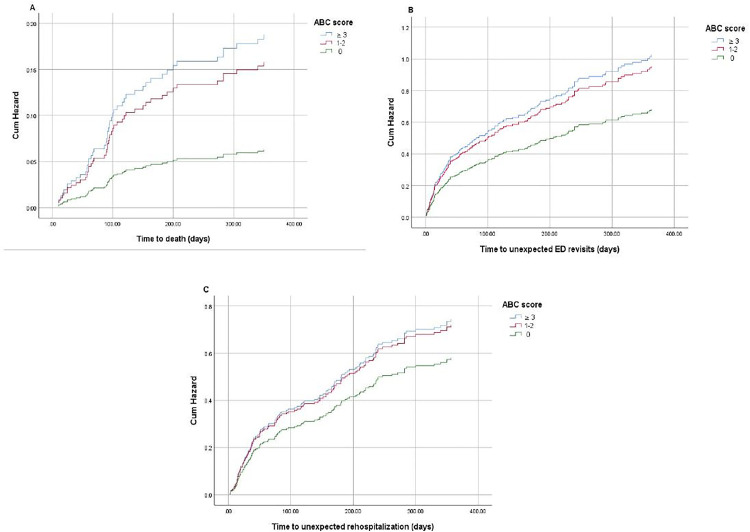
Multivariate analysis of outcomes. Multivariate Cox proportional hazard analysis of anticholinergic burden on clinical outcomes after discharge. A: all-cause mortality, B: unexpected ED revisit, and C: unexpected rehospitalization (adjusted by age, sex, CCI, NAF, MoCA, CFS scores, and number of prescribed medicines).

We further performed a multivariable Cox proportional hazard analysis of patients with an ACB score ≥ 3 compared to those with lower ACB scores regarding adverse clinical outcomes, as shown in Fig 2. The multivariable Cox model was adjusted for potential factors for adverse clinical outcomes such as age, sex, number of prescribed medications, and CFS, MoCA, NAF, and CCI scores. The ACB score ≥ 3 was slightly associated with mortality during the one year after discharge (HR 2.98, 95% CI 0.96–9.28; p = 0.059). However, the exposure to an ACB score ≥ 3 was not a greater risk of unexpected rehospitalization, and ED revisit during one year after discharge (HR 1.28, 95% CI 0.73–2.24; p = 0.384 and HR 1.50, 95% CI 0.91–2.49; p = 0.115, respectively)

## Discussion

The prospective cohort study evaluated the impact of anticholinergic burden on clinical outcomes among older patients one year after discharge. We also determined the incidence rates of adverse clinical outcomes based on the severity of the anticholinergic burden in one year after discharge. Additionally, the study reported changes in the total ACB score from admission to discharge. In our study, more than half of older patients at discharge were prescribed medications with anticholinergic properties, which is consistent with the findings of Lattanzio et al. [11]. Lattanzio et al. reported that 68.3% of hospitalized patients aged ≥ 65 years in acute care wards received anticholinergic medications at discharge as determined by the ACB scale [11]. Furthermore, 37.9% of the participants experienced a high anticholinergic burden, a significantly higher prevalence than that reported by Wilczyński et al. (13.68%) and Pfistermeister et al. (12.1%) [15,17]. In almost 50% of cases, the ACB score increased from admission to discharge. Notably, Herrero-Zazo et al. [16] reported a decrease in the anticholinergic burden from admission to discharge when geriatricians were involved in patient care.

In contrast to previous studies, characteristics such as age, sex, educational level, and number of comorbidities did not differ between the groups of patients with different ACB scores [20,27,28,46,47]. Regarding the utilization of healthcare services, our findings are consistent with those of Reppas-Rindlisbacher et al. [48], who demonstrated that patients who met multiple physicians and had numerous visits were prone to a high anticholinergic burden. Nevertheless, we did not find a relationship between an increased LOS and a high ACB score, contrary to the findings of other studies [49–54].

In terms of comorbidities, higher prevalence of dementia, congestive heart failure, and anemia were found in patients with higher ACB scores. According to the study by O’Dwyer et al. [55], older patients with intellectual disabilities, including dementia, have a higher anticholinergic burden and are more likely to use various types of anticholinergic medications, including antipsychotics and benzodiazepine, than those without intellectual disabilities. As expected, patients with cardiovascular diseases such as heart failure were prescribed more medications with anticholinergic activities, for example, diuretics, vasodilators, and beta-blockers, than those without cardiovascular disease [56,57]. Furthermore, anticholinergic medications may cause anemia through several mechanisms, primarily by inhibiting iron absorption in the stomach. In addition, anticholinergic medications increase erythrocyte turnover via M1 muscarinic receptors on erythrocytes and bone marrow [58–61].

With regard to cognitive, psychological, and functional status, there were no significant differences in the patient’s cognitive function, malnutrition, or depressive symptoms among the groups with different ABC scores, contrasting with recent studies [15,17,62–65].

As demonstrated in this study, patients with high ACB scores also experienced polypharmacy, including the use of benzodiazepines, antipsychotics, antidepressants, muscle relaxants, antihistamines, and antiparkinsonian medications, which aligns with other studies [27–29]. These medications, such as benzodiazepines and antihistamines, are well-known to have anticholinergic activity. In this study, the most common class of anticholinergic medication prescribed at discharge was benzodiazepine, which may be linked to cognitive impairment [66–69]. Although benzodiazepines are included in the ACB scale with a score of 1, this reflects possible anticholinergic activity without established cognitive adverse effects directly related to anticholinergic mechanisms. The cognitive impairment often associated with benzodiazepines may be due to their central GABAergic effects and glutamate pathway inhibitions rather than anticholinergic properties per se [70]. Besides, it has been found that corticosteroids were among the most frequently prescribed anticholinergic medications both at admission and discharge for treatment of chronic conditions such as malignancy with complications and autoimmune diseases. Although corticosteroids have scored 1 in the ACB scale, the cognitive decline related to corticosteroids has been explained by the mechanism of increased neuronal vulnerability to apoptosis and hippocampal atrophy [71]. On the contrary, furosemide was the most commonly prescribed anticholinergic medication in studies conducted by Kidd et al. and Lattanzio et al. [11,49].

In the study, the mortality rate one year after discharge of older patients exposed to a high anticholinergic burden was 20%, which is higher than that reported by Gutiérrez-Valencia et al. [72]. This could be explained by the different population settings, anticholinergic burden scales, and follow-up periods used in the studies. After adjusted by the multivariable Cox proportional hazard model for potential factors of negative clinical outcomes such as age, sex, number of prescribed medications, and CFS, MoCA, NAF, and CCI scores, there was no significant association between an increased ACB score and the risk of adverse clinical outcomes, including all-cause mortality, falls, unexpected rehospitalization, and ED revisits within one year after discharge. This may be attributable to the absence of several potential confounding factors, including post-discharge medication adherence, the use of non-prescribed anticholinergic agents with anticholinergic properties, and cognitive function after hospital discharge, which could significantly influence the outcome. However, in two large cohort studies, a higher ACB score was found to correlate with mortality over follow-up periods of 14.9 years [44] and 2 years [73]. This may be explained by the longer follow-up periods showing the relationship between mortality and high ACB scores. In addition, in contrast to our study, previous studies have shown that a higher ACB score is associated with increased gait and balance problems and fall-related injuries [74,75]. It has also been reported that anticholinergic medications are associated with a higher risk of falls and fractures due to their central adverse effects, which include sedation, dizziness, and confusion [76–78].

### Strengths and limitations

To our knowledge, this study represents the impact of an anticholinergic burden on clinical outcomes such as mortality, unplanned rehospitalization, ED revisits, and falls within the context of Thailand. In addition, the study demonstrated changes in anticholinergic medications use from admission to discharge. The key strength of our study is the comprehensive data retrieved from clinical and functional assessments, structured interview-administered questionnaires, and EMRs. Trained assessors conducted clinical and functional evaluations, including cognitive evaluation, nutritional and frailty status, and activities of daily living. Furthermore, the anticholinergic burden was evaluated and calculated using a standardized and validated ACB scale by trained medical personnel. However, several limitations have to be consideration. First, the study did not take into account the prescribed dose and duration of the anticholinergic medications, which may have resulted in an underestimation of their influence on negative clinical outcomes. Second, changes in anticholinergic medication use and ACB scores after discharge were neither documented nor taken into account. These factors could impact clinical outcomes after discharge. Finally, the study does not account for several potential confounding factors that may significantly influence the outcomes, such as mortality, unexpected ED visits, and rehospitalization. These include post-discharge medication adherence, the use of non-prescribed anticholinergic agents (over-the-counter medications with anticholinergic properties), and cognitive function after hospital discharge.

### Implications

Hospital physicians and clinical pharmacists should conduct continuous medication reconciliation and review from admission through discharge, with a focus on identifying inappropriate anticholinergic medications. This process should incorporate the application of explicit criteria, such as the Beers [79] and STOPP criteria [80], along with anticholinergic burden assessment tools, including the ACB score [43], as part of routine geriatric care. Inappropriate anticholinergic medications should be discontinued through a structured trial of deprescribing [81], particularly in cases of long-term use, to minimize the risk of adverse withdrawal effects. In addition, physicians and clinical pharmacists should acknowledge the potential adverse consequences associated with benzodiazepines, which represent the most frequently prescribed anticholinergic agents. In older adults, these risks include cognitive impairment that may arise not only from their anticholinergic properties but also from central GABAergic effects. Furthermore, nursing staff could be supported to engage in proactive monitoring for signs and symptoms suggestive of adverse events.

## Conclusion

One-third of older patients were exposed to a high anticholinergic burden upon admission, and more than half of the participants had a higher anticholinergic burden at discharge than at admission. The incidence rate of all-cause mortality in one year after discharge among hospitalized older patients with a high anticholinergic burden (ACB score ≥ 3) was 0.65 cases per 1000 person-days. In addition, a higher ACB score at discharge may indicate an increased risk of mortality among older patients discharged from acute care settings.

## Supporting information

S1 Table The list of drugs with the ACB score. (DOCX)

S2 Table Geriatric conditions among hospitalized older patients stratified by the total ACB score. (DOCX)

S3 Table The list of drugs with the ACB score at admission. (DOCX)

S4 Table The list of drugs with the ACB score at discharge. (DOCX)

S5 Table Biochemical profiles among hospitalized older patients stratified by the total ACB score. (DOCX)

S1 Data Raw data. (XLSX)

## Abbreviations

ACB: Anticholinergic Cognitive Burden
EMR: Electronic Medical Record
CFS: Clinical Frailty Scale
CNS: Central Nervous System
NAF: Nutritional Alert Form
MoCA: Montreal Cognitive Assessment
TGDS-15: Thai Geriatric Depression Scale-15
BMI: Body Mass Index
LOS: Length of Hospital Stay
ADL: Activity of Daily Living
IADL: Instrumental Activity of Daily Living
ED: Emergency Department
OPD: Out Patient Department
COPD: Chronic Obstructive Pulmonary Disease
ICU: Intensive Care Unit
ATC: Anatomical Therapeutic Chemical
CCI: Charlson Comorbidity Index
