# Supplementary material for: Anticholinergic burden and clinical outcomes among older adults admitted in a tertiary hospital: a prospective cohort study
Source: PLoS One. 2025 Sep 19;20(9):e0332946. doi: 10.1371/journal.pone.0332946 (PMC12448347; doi:10.1371/journal.pone.0332946)
Supplement: S1 Table — (DOCX) [file pone.0332946.s001.docx]

**S1 Table. The list of drugs with the ACB score**

| **Drugs with ACB Score of 1** | **Drugs with ACB Score of 2** | **Drugs with ACB Score of 3** |
| --- | --- | --- |
| Alprazolam | Carbamazepine | Amitriptyline |
| Aripiprazole | Perphenazine | Chlorpheniramine |
| Atenolol | Tramadol | Cyproheptadine |
| Cetirizine |  | Dimenhydrinate |
| Clonazepam |  | Hydroxyzine |
| Codeine |  | Olanzapine |
| Colchicine |  | Orphenadrine |
| Desloratadine |  | Quetiapine |
| Dexamethasone |  | Trospium |
| Dextromethorphan |  |  |
| Diazepam |  |  |
| Digoxin |  |  |
| Escitalopram |  |  |
| Fentanyl |  |  |
| Fluoxetine |  |  |
| Hydralazine |  |  |
| Isosorbide dinitrate |  |  |
| Levocetirizine |  |  |
| Levodopa |  |  |
| Loperamide |  |  |
| Loratadine |  |  |
| Lorazepam |  |  |
| Metformin |  |  |
| Metoprolol |  |  |
| Midazolam |  |  |
| Mirtazapine |  |  |
| Morphine |  |  |
| Nifedipine |  |  |
| Prednisolone |  |  |
| Risperidone |  |  |
| Sertraline |  |  |
| Theophylline |  |  |
| Trazodone |  |  |
| Valproic acid |  |  |
| Venlafaxine |  |  |
| Warfarin |  |  |
