# Supplementary material for: Anticholinergic burden and clinical outcomes among older adults admitted in a tertiary hospital: a prospective cohort study
Source: PLoS One. 2025 Sep 19;20(9):e0332946. doi: 10.1371/journal.pone.0332946 (PMC12448347; doi:10.1371/journal.pone.0332946)
Supplement: S2 Table — (DOCX) [file pone.0332946.s002.docx]

**S2 Table. Geriatric conditions among hospitalized older patients stratified by the total ACB score**

| Characteristics | Total  (n=290)  N (%) | ACB score at discharge | | | |
| --- | --- | --- | --- | --- | --- |
|  |  | ACB score 0 N=52  (18.0%)  N (%) | ACB score 1-2  N=128  (44.1%)  N (%) | ACB score ≥ 3  N=110  (37.9%)  N (%) | *P* value |
| **Geriatric conditions** |  |  |  |  |  |
| MoCA score, median (IQR) | 16 (11, 21) | 17 (11.5, 21) | 16 (11, 22) | 15 (10, 20) | 0.378^+^ |
| MoCA score < 25 | 250 (86.2) | 45 (86.5) | 108 (84.4) | 97 (88.2) | 0.695^*^ |
| TGDS score, median (IQR) | 0 (0, 2) | 0 (0, 1) | 0 (0, 2) | 0 (0, 2) | 0.111^+^ |
| TGDS score > 5 | 17 (5.9) | 1 (1.9) | 10 (7.8) | 6 (5.5) | 0.305^*^ |
| BADLs, mean (SD) | 18.3 (2.7) | 18.8 (2.1) | 18.1 (2.9) | 18.3 (2.7) | 0.239^#^ |
| BADL impairment | 7 (2.4) | 1 (1.9) | 3 (2.3) | 3 (2.7) | 1.000^*^ |
| IADLs, median (IQR) | 6 (1, 8) | 7 (4, 8) | 6 (1, 8) | 5 (1, 8) | 0.021^+^ |
| IADL impairment | 183 (63.1) | 27 (51.9) | 78 (60.9) | 78 (70.9) | 0.052^*^ |
| CFS score, mean (SD) | 3.8 (1.2) | 3.5 (1.2) | 3.8 (1.2) | 3.9 (1.3) | 0.150^#^ |
| CFS score ≥ 5 | 68 (23.4) | 8 (15.4) | 31 (24.2) | 29 (26.4) | 0.294^*^ |
| NAF score, median (IQR) | 7 (4, 11) | 6 (3.25, 10) | 6.5 (5, 11) | 7 (5, 11) | 0.607^+^ |
| NAF score >5 | 202 (69.7) | 33 (63.5) | 91 (71.1) | 78 (70.9) | 0.562^*^ |
| Sleep disorder | 125 (43.1) | 16 (30.8) | 54 (42.2) | 55 (50.0) | 0.067^*^ |
| Urinary incontinence | 10 (3.4) | 2 (3.8) | 4 (3.1) | 4 (3.6) | 1.000^*^ |
| Fecal incontinence | 7 (2.4) | 1 (1.9) | 2 (1.6) | 4 (3.6) | 0.690^*^ |
| History of fall in 3 month | 15 (5.2) | 1 (1.9) | 6 (4.7) | 8 (7.3) | 0.338^*^ |
| History of depression | 15 (5.2) | 3 (5.8) | 8 (6.3) | 4 (3.6) | 0.647^*^ |
| Pressure ulcer | 12 (4.1) | 0 (0) | 6 (4.7) | 6 (5.5) | 0.264^*^ |
| Visual impairment | 19 (6.6) | 3 (5.8) | 10 (7.8) | 6 (5.5) | 0.741^*^ |
| Hearing impairment | 32 (11.0) | 2 (3.8) | 18 (14.1) | 12 (10.9) | 0.140^*^ |
| Significant weight loss | 37 (12.8) | 7 (13.5) | 14 (10.9) | 16 (14.5) | 0.698^*^ |
| Artificial enteral nutrition | 9 (3.1) | 1 (1.9) | 5 (3.9) | 3 (2.7) | 0.823^*^ |
| Pain | 22 (7.6) | 6 (11.5) | 8 (6.3) | 8 (7.3) | 0.472^*^ |
| Ambulation with assistance | 113 (39.0) | 15 (28.8) | 54 (42.2) | 44 (40.0) | 0.241^*^ |
| Body mass index (kg/m^2^), mean (SD) | 23.1 (4.6) | 23.4 (3.9) | 23.1 (4.7) | 23.0 (4.7) | 0.866^#^ |
| Body weight (kg), mean (SD) | 59.4 (13.2) | 59.8 (12.1) | 59.5 (13.1) | 59.0 (13.9) | 0.924^#^ |

**Data are presented as** mean (standard deviation), n (%), or median (interquartile range)

^*^ Chi-square test, ^#^ Student’s t-test, ^+^ Mann–Whitney U test

**Abbreviations:** IQR, interquartile range; SD, standard deviation; NAF, Nutritional Alert Form; MoCA, Montreal Cognitive Assessment; TGDS, Thai Geriatric Depression Scale; CFS, Clinical Frailty Scale; BADL, Basic activities of daily living; IADL, Instrumental activities of daily living; kg, kilogram, m, meter; ACB, anticholinergic cognitive burden
