# Supplementary material for: Anticholinergic burden and clinical outcomes among older adults admitted in a tertiary hospital: a prospective cohort study
Source: PLoS One. 2025 Sep 19;20(9):e0332946. doi: 10.1371/journal.pone.0332946 (PMC12448347; doi:10.1371/journal.pone.0332946)
Supplement: S3 Table — (DOCX) [file pone.0332946.s003.docx]

**S3 Table. The list of drugs with the ACB score at admission**

| **Drugs with ACB Score of 1** | **N (all) = 244**  **N (%)** | **Drugs with ACB Score of 2** | **N (all) = 244**  **N (%)** |
| --- | --- | --- | --- |
| Alprazolam | 1 (0.4) | Carbamazepine | 1 (0.4) |
| Aripiprazole | 2 (0.8) | Perphenazine | 0 (0) |
| Atenolol | 7 (2.9) | Tramadol | 7 (2.9) |
| Cetirizine | 12 (4.9) | **Drugs with ACB Score of 3** |  |
| Clonazepam | 7 (2.9) | Amitriptyline | 0 (0) |
| Codeine | 1 (0.4) | Chlorpheniramine | 2 (0.8) |
| Colchicine | 3 (1.2) | Cyproheptadine | 2 (0.8) |
| Desloratadine | 0 (0) | Dimenhydrinate | 3 (1.2) |
| Dexamethasone | 7 (2.9) | Hydroxyzine | 1 (0.4) |
| Dextromethorphan | 5 (2.0) | Olanzapine | 3 (1.2) |
| Diazepam | 1 (0.4) | Orphenadrine | 3 (1.2) |
| Digoxin | 3 (1.2) | Quetiapine | 14 (5.7) |
| Escitalopram | 3 (1.2) | Trospium | 1 (0.4) |
| Fentanyl | 2 (0.8) |  |  |
| Fluoxetine | 1 (0.4) |  |  |
| Hydralazine | 23 (9.4) |  |  |
| Isosorbide dinitrate | 15 (6.1) |  |  |
| Levocetirizine | 2 (0.8) |  |  |
| Levodopa | 2 (0.8) |  |  |
| Loperamide | 1 (0.4) |  |  |
| Loratadine | 3 (1.2) |  |  |
| Lorazepam | 27 (11.1) |  |  |
| Metformin | 6 (2.5) |  |  |
| Metoprolol | 6 (2.5) |  |  |
| Midazolam | 2 (0.8) |  |  |
| Mirtazapine | 1 (0.4) |  |  |
| Morphine | 8 (3.3) |  |  |
| Nifedipine | 2 (0.8) |  |  |
| Prednisolone | 33 (13.5) |  |  |
| Risperidone | 2 (0.8) |  |  |
| Sertraline | 5 (2.0) |  |  |
| Theophylline | 1 (0.4) |  |  |
| Trazodone | 7 (2.9) |  |  |
| Valproic acid | 1 (0.4) |  |  |
| Venlafaxine | 0 (0) |  |  |
| Warfarin | 5 (2.0) |  |  |
