# Supplementary material for: Anticholinergic burden and clinical outcomes among older adults admitted in a tertiary hospital: a prospective cohort study
Source: PLoS One. 2025 Sep 19;20(9):e0332946. doi: 10.1371/journal.pone.0332946 (PMC12448347; doi:10.1371/journal.pone.0332946)
Supplement: S4 Table — (DOCX) [file pone.0332946.s004.docx]

**S4 Table. The list of drugs with the ACB score at discharge**

| **Drugs with ACB Score of 1** | **N (all) = 430**  **N (%)** | **Drugs with ACB Score of 2** | **N (all) = 430**  **N (%)** |
| --- | --- | --- | --- |
| Alprazolam | 2 (0.5) | Carbamazepine | 0 (0) |
| Aripiprazole | 2 (0.5) | Perphenazine | 1 (0.2) |
| Atenolol | 7 (1.6) | Tramadol | 24 (5.6) |
| Cetirizine | 17 (3.9) | **Drugs with ACB Score of 3** |  |
| Clonazepam | 9 (2.1) | Amitriptyline | 2 (0.5) |
| Codeine | 1 (0.2) | Chlorpheniramine | 0 (0) |
| Colchicine | 15 (3.5) | Cyproheptadine | 2 (0.5) |
| Desloratadine | 2 (0.5) | Dimenhydrinate | 4 (0.9) |
| Dexamethasone | 8 (1.9) | Hydroxyzine | 2 (0.5) |
| Dextromethorphan | 10 (2.3) | Olanzapine | 2 (0.5) |
| Diazepam | 1 (0.2) | Orphenadrine | 9 (2.1) |
| Digoxin | 5 (1.2) | Quetiapine | 25 (5.8) |
| Escitalopram | 4 (0.9) | Trospium | 1 (0.2) |
| Fentanyl | 3 (0.7) |  |  |
| Fluoxetine | 1 (0.2) |  |  |
| Hydralazine | 20 (4.7) |  |  |
| Isosorbide dinitrate | 24 (5.6) |  |  |
| Levocetirizine | 4 (0.9) |  |  |
| Levodopa | 2 (0.5) |  |  |
| Loperamide | 3 (0.7) |  |  |
| Loratadine | 2 (0.5) |  |  |
| Lorazepam | 58 (13.5) |  |  |
| Metformin | 40 (9.3) |  |  |
| Metoprolol | 8 (1.9) |  |  |
| Midazolam | 0 (0) |  |  |
| Mirtazapine | 4 (0.9) |  |  |
| Morphine | 13 (3.0) |  |  |
| Nifedipine | 1 (0.2) |  |  |
| Prednisolone | 50 (11.6) |  |  |
| Risperidone | 4 (0.9) |  |  |
| Sertraline | 6 (1.4) |  |  |
| Theophylline | 0 (0) |  |  |
| Trazodone | 9 (2.1) |  |  |
| Valproic acid | 2 (0.5) |  |  |
| Venlafaxine | 1 (0.2) |  |  |
| Warfarin | 20 (4.7) |  |  |
