# Supplementary material for: Anticholinergic burden and clinical outcomes among older adults admitted in a tertiary hospital: a prospective cohort study
Source: PLoS One. 2025 Sep 19;20(9):e0332946. doi: 10.1371/journal.pone.0332946 (PMC12448347; doi:10.1371/journal.pone.0332946)
Supplement: S5 Table — (DOCX) [file pone.0332946.s005.docx]

**S5 Table. Biochemical profiles among hospitalized older patients stratified by the total ACB score**

| Characteristics | Total  (n=290)  N (%) | ACB score at discharge | | | |
| --- | --- | --- | --- | --- | --- |
|  |  | ACB score 0 N=52  (18.0%)  N (%) | ACB score 1-2  N=128  (44.1%)  N (%) | ACB score ≥ 3  N=110  (37.9%)  N (%) | *P* value |
| White blood cell count (cells/mm^3^), median (IQR) | 8385  (5687, 12657) | 8465  (6645, 13125) | 9020  (5570, 12840) | 8090  (5500, 12390) | 0.507^+^ |
| Hemoglobin (g/dL), mean (SD) | 10.6 (2.6) | 10.8 (2.8) | 10.6 (2.6) | 10.6 (2.4) | 0.894^#^ |
| Blood urea nitrogen (mg/dL),  median (IQR) | 19.5 (14, 33) | 20 (14, 32.5) | 21 (14, 34) | 18.5 (14, 31.5) | 0.275^+^ |
| Creatinine (mg/dL), median (IQR) | 1.2 (0.8, 1.9) | 1.1 (0.8, 1.3) | 1.2 (0.8, 1.8) | 1.1 (0.8, 1.9) | 0.225^+^ |
| eGFR (mL/min/1.73m^2^), median (IQR) | 58  (30.8, 82.8) | 65.6  (44.3, 78.9) | 51.9  (35.3, 88.0) | 59.4  (26.6, 82.5) | 0.191^+^ |
| Sodium (mmol/L), mean (SD) | 135.0 (6.6) | 135.2 (7.8) | 134.5 (6.8) | 135.6 (5.7) | 0.405^#^ |
| Potassium (mmol/L), mean (SD) | 4.1 (0.6) | 4.2 (0.6) | 4.0 (0.6) | 4.1 (0.7) | 0.347^#^ |
| Calcium (mg/dL), median (IQR) | 8.9 (8.4, 9.4) | 8.9 (8.4, 9.2) | 8.9 (8.3, 9.3) | 8.9 (8.5, 9.4) | 0.473^+^ |
| Magnesium (mg/dL), median (IQR) | 1.9 (1.7, 2.1) | 1.9(1.8, 2.1) | 1.9 (1.7, 2.1) | 1.9 (1.7, 2.1) | 0.678^+^ |
| Phosphate (mg/dL), median (IQR) | 3.2 (2.6, 3.8) | 3.1 (2.6, 3.5) | 3.2 (2.5, 3.6) | 3.2 (2.6, 4.1) | 0.565^+^ |
| Aspartate aminotransferase (U/L),  median (IQR) | 35.5 (26, 54.3) | 34 (26, 60) | 32 (24, 44) | 32 (25, 48) | 0.654^+^ |
| Alanine aminotransferase (U/L), median (IQR) | 23 (13, 47) | 19 (13, 58.5) | 18.5 (12, 34) | 21 (11.5,38.5) | 0.842^+^ |
| Alkaline phosphatase (U/L),  median (IQR) | 94.5  (69, 132.3) | 99  (62, 124.5) | 91.5  (65, 126) | 95.5  (77, 136.5) | 0.810^+^ |
| Total bilirubin (mg/dL), median (IQR) | 0.7 (0.5, 1.1) | 0.8 (0.5, 1.2) | 0.7 (0.5, 1.3) | 0.7 (0.5, 1.0) | 0.854^+^ |
| Albumin (g/L), mean (SD) | 31.1 (6.9) | 32.2 (6.1) | 30.9 (6.9) | 30.8 (7.2) | 0.483^#^ |
| Blood sugar (mg/dL), median (IQR) | 129  (109, 161.5) | 125  (104.5, 150.5) | 135  (112, 172) | 128  (109, 160) | 0.321^+^ |

**Data are presented as** mean (standard deviation) or median (interquartile range)

^#^ Student’s t-test, ^+^ Mann–Whitney U test

**Abbreviations:** mm, millimeter; g, gram; dL, deciliter; mg, milligram; mL, milliliter; mmol, millimole; L, liter; U, unit; SD, standard deviation; IQR, interquartile range; ACB, anticholinergic cognitive burden
